# The association between reactive strength index and reactive strength index modified with approach jump performance

**DOI:** 10.1371/journal.pone.0264144

**Published:** 2022-02-17

**Authors:** Jernej Pleša, Žiga Kozinc, Darjan Smajla, Nejc Šarabon

**Affiliations:** 1 Faculty of Health Sciences, University of Primorska, Izola, Slovenia; 2 Andrej Marušič Institute, University of Primorska, Koper, Slovenia; 3 InnoRenew CoE, Human Health Department, Izola, Slovenia; 4 S2P, Science to Practice, Ltd., Laboratory for Motor Control and Motor Behavior, Ljubljana, Slovenia; Universita degli Studi di Verona, ITALY

## Abstract

Jumping performance is one of the key components of volleyball game, thus evaluating jumping ability through different biomechanical variables offers opportunity for performance optimization. The aim of this study was to assess the associations between reactive strength index (RSI), reactive strength index modified (RSI_mod_) and approach jump performance in male volleyball players. Forty volleyball players performed drop jump (DJ) form 40 cm high box, bilateral and unilateral countermovement jumps (CMJ) and approach jump. RSI in DJ was calculated as the ratio between jump height and ground contact time, while the RSI in CMJ tasks (RSI_mod_) was calculated as ratio between jump height and jump time. Our results indicate that the relationships among different RSI variants and approach jump in volleyball players are moderate to strong (r = 0.42–0.73), with the highest correlations being observed for RSI_mod_ from bilateral CMJ (r = 0.676–0.727). Those observations are in line with the principle of movement specificity, which suggests that the best performance indicator should be the task that best resembles the demands of the sport-specific movements. Further research is needed to reveal more about the potential of implementing these findings for training optimization through monitoring RSI and RSI_mod_ values.

## 1 Introduction

Vertical jumping is one of the most important physical capabilities for successful volleyball performance [1]. The higher a player is able to jump, the greater his/her potential for successful performance in offensive and defensive actions [2]. The monitoring of various performance characteristics of athletes is crucial component in strength and conditioning practice. Approach jump performance is one of the sport-specific tasks in volleyball gameplay [3, 4], which warrants exploration of its underlying biomechanical determinants that could be used for training optimization.

Jumping ability is evaluated through different forms (unilateral and bilateral, vertical and horizontal) with the use of different measurement devices [5]. In volleyball practice, approach jump is usually performed for evaluating athletic-specific performance of volleyball players [6]. Approach jump is characterized by countermovement with the use of arm movement and presents a combination of a drop jump (DJ) and a countermovement jump (CMJ) [5]. Players normally use 2- to 3-step approach [5], with an explosive penultimate step [7]. Penultimate step is also called the approach phase, which is defined between the last step take-off and the ground contact of both feet, followed by plant phase which lasts from ground contact of both feet to take-off [8]. Penultimate step could be also presented as a half-drop jump [9], followed by a countermovement arm swing and an eccentric contraction that exploits the stretch-shortening cycle (SSC) of the activated muscles [5, 10]. In push off phase, one leg is usually in front of the other, with foot directed slightly inwards, to emphasize vertical direction of the jump and prevent too much horizontal flight [11, 12]. The purpose of approach is to create high horizontal force, which is later transferred into vertical direction to jump as high as possible [7]. Apporach jump presents the hight at which volleyball player can spike the ball in the attacking action, while the difference between standing reach and jumping reach presents the height of the jump [5].

With more analytical approach and biomechanical testing, we can evaluate jumping characteristics throughout more complex forms of performance tests. Those tests provide a more detailed insight into the athlete’s neuromuscular capacity and thus offer the opportunity for training optimization. Previous research has identified the reactive strength index (RSI) as a variable that can be used to assess an athlete’s reactive strength [13]. Reactive strength is the ability to rapidly and efficiently transition from an eccentric to a concentric muscle contraction within a SSC movement [14]. The SSC is present during many sporting activities, such as sprinting and jumping [15, 16]. Activities such as sprinting and jumping largely depend on the ability to develop maximal force in a minimal bout of time [17]. The RSI is a measure of produced force and the time to develop this force, which is calculated as the ratio between jump height and ground contact time [13], thereby assessing vertical reactive strength [18] and may present potentially useful tool for designing individually tailored plyometric training [19]. Recent studies indicated that modified version of RSI (RSI_mod_), obtained from counter-movement jump (CMJ) metrics, may provide an alternative method for assessing RSI during several different plyometric exercises [20]. Calculation of RSI_mod_ is similar to that of RSI, with groud contact time (DJ) being replaced with the time to takeoff (CMJ). Similar to RSI [21], the RSI_mod_ is considered a reliable measure and was reported to discriminated between different groups of athletes [22]. The RSI in DJ is considered to represent fast SSC ability (ground contact < 250 ms) [23], whereas the RSI_mod_ represents slow SSC ability. Therefore, both RSI and RSI_mod_ could present useful method for evaluation of jumping characteristics of volleyball players.

The purpose of this study was to examine the relationship between RSI and RSI_mod_ (from both bilateral and unilateral CMJ) in different tasks with approach jump performance in volleyball players. Approach jump performance is one of the key components for successful volleyball performance [3, 4], thus its association with RSI could present potentially useful tool to guide training-related decision making for improving jumping performance. We hypothesized that all RSI outcomes will be positively related to approach jump height.

## 2 Methods

### 2.1 Subjects

For this study, we recruited 40 male volleyball players (age: 20.3 ± 3.3 years; body height: 187.4 ± 7.75 cm; body mass: 79.2 ± 8.6 kg). All the players have been competing in 1^st^ or 2^nd^ division of the national league. They reported to be involved in regular training for 10.9 ± 4.1 years, to attend 5.7 ± 1.2 training sessions per week and to regularly perform full body resistance exercises at least twice a week. The exclusion criteria were the presence of musculoskeletal injuries in the previous 6 months. The participants were informed about the experimental procedures and were required to sign an informed consent before participating in the experiment. For underage participants, their parents or legal guardians signed the consent on their behalf. The experiment was approved by Republic of Slovenia National Medical Ethics Committee (approval no. 0120–99/2018/5) and was conducted in accordance to the latest revision of the Declaration of Helsinki.

### 2.2 Study design

This was a cross-sectional study, with all measurements conducted in a single visit. The participants had been performing bilateral and unilateral jumps as part of their regular training and assessments. The participants performed a warm-up consisting of 10 min of self-pace jogging, 5 min of dynamic stretching and 5 min of bodyweight resistance exercises (squats, lunges, push-ups) and 3 min of activation exercises (vertical jumps and short-distance sprints). Then, they completed assessments of vertical jumps on a force plate (DJ, bilateral CMJ and unilateral CMJ) and volleyball specific performance test (approach jump). The order of the tasks was randomized across participants. For all tasks, three trials were performed, and the average of the three trials was taken for further analysis.

### 2.3 Assessment of drop jump and countermovement jump tests

DJs and CMJs were performed on a piezoelectric force plate (Kistler, model 9260AA6, Winterthur, Switzerland). Ground reaction force data were recorded at sampling rate of 1000 Hz. The signals were automatically processed by the manufacturer’s software (MARS, Kistler, Winterthur, Switzerland) by a moving average filter with a 5 ms window. Participants performed three warm-up trials for each jumping task. Each task was performed three times, with a 60-s rest between trials. The hands were placed on the hips at all times. For the DJ, the participants stood on a solid 40 cm high box, which was shown to reflect the highest and most reliable RSI in the population of professional basketball players [24]. The participants stepped off the box and performed a vertical jump immediately after the landing. They were instructed to achieve maximal jump height whilst minimizing the ground contact time. When performing the DJ participants maintained upright posture. The jump height was calculated based on take-off velocity. Contact times were also taken for the analysis, and subsequently, the RSI (RSI_DJ_) was calculated as the ratio between the jump height and the contact time. Ground contact time was defined as the time during which the force signal was > 10 N.

When performing CMJ, the participants were instructed to start from the standing position and use an explosive countermovement to a self-selected depth and to jump as high as possible. Self-selected depth for performing CMJ was chosen as it was shown to be superior for jump height and RSI_mod_ values [25]. For the unilateral CMJ, the non-tested leg was slightly flexed at the knee and was not allowed to touch the tested leg. Performing the swing with the non-tested leg was not allowed. Participants performed three repetitions for each leg in an alternating order, with 1-minute breaks between the repetitions. The jump height was calculated based on take-off velocity. RSI was calculated by dividing the jump height with the time to take off. Time to take off was determined as the time between the countermovement initiation (defined as the decrease in force signal larger than 3 standard deviations of the baseline signal) and the take-off (defined as the first instant of force < 10 N), which was shown to be reliable method to determine the take-of instant [26].

### 2.4 Assessment of volleyball specific approach jump

Basketball board with measurement tape was used to record approach jump heights. Before jumping attempts, participants chalked their fingertips for more precise detection of the jumping reach. Jumping height was calculated as a difference between standing reach and jumping reach. All the participants were experienced volleyball players, so the only instruction was to jump as high as possible, by utilizing a normal spike approach. All the participants were experienced volleyball players, so further standardization could have negative impact on jumping performance. Each participant performed two warm-up trials at submaximal effort and three testing attempts, with 1-min breaks in between. Measurements were read to the nearest centimeter.

### 2.5 Statistical analysis

The data were analyzed with SPSS (version 25.0, SPSS Inc., Chicago, USA). Descriptive statistics are reported as mean ± standard deviation, minimum and maximum values. The normality of the data distribution was checked with Shapiro-Wilk tests. Trial-to-trial reliability was assessed with two way random, single measures, absolute agreement model for calculating intra-class correlation coefficient (ICC) and typical error, expressed relative to the mean (coefficient of variation; CV). Correlations between different RSI outcomes and approach jump performance were assessed with Pearson’s correlation coefficients and interpreted as negligible (< 0.1), weak (0.1–0.4), moderate (0.4–0.7), strong (0.7–0.9) and very strong (> 0.9) [27]. The threshold for statistical significance was set at p < 0.05.

## 3 Results

The descriptive statistics for all variables are presented in Table 1. The correlations among RSI variables and approach jump performance are presented in Table 2. The approach jump showed excellent reliability (ICC = 0.99; CV = 2.45%). All jump height outcomes also showed excellent reliability (ICC = 0.93–0.96; CV = 4.7–6.5%). The RSI and all RSI_mod_ outcomes had excellent relative reliability (ICC = 0.91–0.93). RSI and bilateral RSI_mod_ also had acceptable absolute reliability (CV = 6.5 and 6.8%), while the absolute reliability for the unilateral RSI_mod_ was on the border of acceptable threshold (CV = 9.6% for the preferred leg and 10.2% for the non-preferred leg).

**Table 1:** Descriptive statistics for all outcome variables.

	Mean	SD	Minimum	Maximum
Approach jump (cm)	78.6	9.47	54.0	96.0
DJ—Jump Height (cm)	0.35	0.07	0.20	0.51
DJ—Contact Time (ms)	0.19	0.03	0.16	0.25
DJ—RSI	1.82	0.44	0.98	2.91
CMJ BL—Jump Height (cm)	0.44	0.06	0.31	0.61
CMJ BL—Jump Time (ms)	0.77	0.09	0.56	0.95
CMJ BL—RSI_mod_	0.58	0.13	0.39	0.97
CMJ D—Jump Height (cm)	0.58	0.05	0.11	0.33
CMJ D—Jump Time (ms)	0.87	0.11	0.69	1.15
CMJ D—RSI_mod_	0.27	0.07	0.15	0.45
CMJ ND–Jump Height (cm)	0.24	0.05	0.12	0.33
CMJ ND–Jump Time (ms)	0.86	0.12	0.68	1.16
CMJ ND—RSI_mod_	0.28	0.07	0.15	0.44

SD–standard deviation; DJ–drop jump; RSI–reactive strength index; CMJ–countermovement jump; BL–bilateral, D–dominant; ND–non dominant.

**Table 2:** Associations between RSI variables and approach jump performance.

	r (with approach jump)	
DJ—Jump Height (cm)		0.42*
DJ—Contact Time (ms)		-0.31
DJ–RSI		0.44**
CMJ BL—Jump Height (cm)		0.73**
CMJ BL—Jump Time (ms)		-0.33*
CMJ BL—RSI_mod_		0.68**
CMJ D—Jump Height (cm)		0.70**
CMJ D—Jump Time (ms)		-0.15
CMJ D—RSI_mod_		0.61**
CMJ ND–Jump Height (cm)		0.73**
CMJ ND–Jump Time (ms)		0.06
CMJ ND—RSI_mod_		0.58**

* p < 0.05;

** p < 0,01; DJ–drop jump; RSI–reactive strength index; CMJ–countermovement jump; BL–bilateral, D–dominant; ND–non dominant.

Approach jump was in moderate correlations with DJ height (r = 0.423; p < 0.05) and RSI (r = 0.436; p < 0.001). In bilateral CMJ, jump height (r = 0.727; p < 0.001) and RSI_mod_ (r = 0.676); p < 0.001) showed high correlations, while jump time showed weak negative correlation (r = -0.331; p < 0.05) with approach jump performance. The negative correlation means that the subjects with shorter jump times jumped higher in approach jump test. In terms of unilateral CMJs (both in dominant and non-dominant side) moderate to high correlations between jump height and RSI_mod_ with approach jump were shown (r = 0.579–0.733; all p < 0.001). Jump time in unilateral CMJs and contact time in DJ were not significantly associated with approach jump performance (p > 0.05). In addition, the RSI was in moderate correlation with all RSI_mod_ variants (r = 0.57–0.70; p < 0.01). Moreover, RSI_mod_ variants were in moderate to high correlation among themselves (r = 0.68–0.78; p < 0.01).

## 4 Discussion

The purpose of this study was to examine the association between RSI and RSI_mod_ with volleyball specific approach jump performance. Our results show that approach jump was a) moderately associated with DJ height and RSI b) moderately to strongly associated with jump height and RSI_mod_ in bilateral CMJ, as well as jump height and RSI_mod_ in both dominant and non-dominant unilateral CMJ. Moreover, the relative reliability of the RSI and bilateral RSI_mod_ were excellent, while the absolute reliability of RSI and bilateral RSI_mod_ were acceptable and in line with previous studies [13, 21]. On the other hand, the absolute reliability of the unilateral RSI_mod_ was on the border of the acceptable treshold. These results suggest that RSI_mod_ could be preferable to RSI when trying to monitor performance and training adaptations in approach jump performance Bilateral variant is suggested to be used in practice due to better absolute reliability.

To our knowledge, this is the first study to date that examined the association between different RSI variants with volleyball specific athletic performance. Our results indicate that the relationships among different RSI variants and approach jump in volleyball players are moderate to strong. Those results are in line with studies reporting associations between approach jump height and CMJ height without arm swing [8] and CMJ with arm swing [28]. Furthermore, a recent systematic review with meta-analysis showed moderate associations between RSI in DJ and independent measures of physical and sporting performance, while the strength of these relationships varied based on the task and physical quality assessed [29]. However, it should be mentioned, that there are some differences in jump characteristics between DJ and CMJ. While in DJ ankle strength and stiffness are main determinants for performance [30], higher contribution of the knee joints is typical for the CMJ [31]. Approach jump is a combination of DJ and CMJ [5], thus positive correlations between approach jump height and both RSI and RSI_mod_ were expected. Furthermore, we observed the highest correlations between bilateral CMJ height and bilateral RSI_mod_ with approach jump height, which is in line with the principle of movement specificity. It is suggested that the best performance indicator should be the task that best resembles the demands of the sport-specific movement task (e.g., unilateral or bilateral actions, horizontally or vertically oriented task). Approach jump is vertically oriented, performed in bilateral circumstances, similar to the characteristics of CMJ.

Higher correlations between approach jump and RSI_mod,_ compared to its correlation with RSI, could be also explained by the specificity of the SSC type. In brief, SSC actions can be roughly classified two different types, fast SSC (contact time < 250 ms) [23], and slow SSC, where the time of descent and transition to ascent is much longer [32]. DJ is performed with the use of fast SSC, while slow SSC is present in exercises such as CMJ [18]. Approach jump characteristics based on the ground contact time and time of downward and upward phase, also reveals the use of slow SSC [8, 33, 34]. Performance enhancement in slow SSC activities may be primarily due to the slow eccentric phase allowing an increased time to develop force [16], while in fast SSC, the underlying mechanisms are based on increased excitability of proprioceptors such as Golgi-tendon organ and muscle spindle [35]. This hypothesis may have implications for volleyball athletic performance training. Different exercises or the manner in which exercises are performed may elicit different mechanisms of SSC action. Moreover, many studies reported the importance of horizontal velocity in the approach phase for jumping performance [8, 28, 36], thus it would be interesting to check the associations between RSI in horizontal direction and approach jump performance. With all of the above in mind, it has to be noted that although RSI outcomes appear to be related to approach jump performance, high correlations between approach jump height and CMJ as well as DJ heights, which means that the RSI offer little additional information. This is probably because approach jump is not a time-restricted task, which means that athletes can take a little longer to perform it (i.e., spend more time on the ground) without compromising the score. Perhaps different results would be observed if we used time-restricted tasks or tasks wherein the performance is defined by time to completion (i.e. sprint). As noted above, RSI in DJ has been shown to be related to several performance proxies [29]. However, further studies should examine independent contributions of RSI and RSI_mod_ to explaining performance in addition to DJ or CMJ height alone in explaining performance.

A common modality to enhance SSC capabilities is plyometric training. Characteristic of plyometric training are quick, powerful movements using a pre-stretch or countermovement that involves SSC [37]. The literature shows that RSI may present potentially useful tool for designing individually-tailored plyometric training [18, 19]. To prescribe plyometric training, the optimal drop height for DJs is suggested to be based on the highest RSI values [38]. The study of Ramirez-Campillo et al. [19] confirmed that statement on sample of young football players, where the group of athletes that performed DJs at highest RSI output exhibit greater performance benefit than the group of athletes performing DJs at the fixed height. Moreover, an intervention study on volleyball players showed that 6-weeks of plyometric training improves jumping ability of volleyball players [39]. Additionally, studies have suggested that RSI_mod_ in bilateral CMJ can be used as a measure of explosiveness in volleyball players [40] and could be used to determine the need of incorporating ballistic-type exercises (i.e., plyometric exercises, weightlifting movements) into an athlete’s training program [41]. Based on the results of this study RSI and RSI_mod_ could potentially present addition insight into neuromuscular characteristics that could be further manipulated with training. For example, for the participants with low RSI values, one of the missing links for better approach jump performance may be bad efficiency of fast stretch shortening cycle. On the other hand, if the athlete has low values of RSI_mod_ this mean low jump height or slow transition from the eccentric to concentric part of the movement. In practice, this would mean that participants with low CMJ height could be directed towards power training, while the participants with slow countermovement transition could be directed towards training improving intermuscular coordination (e.g. rhythmic squats, jumps emphasizing fast transition from countermovement, depth jumps, etc.). Based on the results of this study, RSI and RSI_mod_ could be used as a supplementary part of the training for improving approach jump performance. Nevertheless, it has to be stressed that this metrics should not be used for primary testing of volleyball players, but rather be used as a supplementary diagnostic tool, preferably utilized at the beginning of the season to get a better insight into each individual player.

Some limitations of the study must be acknowledged. The study was conducted on a sample of well-trained male volleyball players. The performance test used in this study covered only a limited aspect of volleyball athletic performance. Referring to RSI, previous studies have found significant differences in RSI among sports and between sexes [41], thus, our results cannot be generalized to other sports and females. Moreover, only DJ task from 40 cm height was included in the study. While some studies reported no differences in RSI computed from DJs of varying heights [42], others suggest that optimal DJ height exists that maximizes RSI [19]. Thus, future studies should consider including multiple DJ with different heights. Finally, the cross-sectional design precludes establishing any causal relationships, thus, further prospective and experimental research is needed to corroborate our results.

## 5 Conclusion

In this study, we found that approach jump performance seems to be associated by both, RSI and RSI_mod_, while the RSI_mod_ exhibit higher correlations with approach jump, thus present preferable method when trying to monitor performance and training adaptations in approach jump performance. Results of this study are in line with the principle of movement specificity, which suggests that the best performance indicator should be the task that best resemble the demands of the sport-specific movements. The literature suggests, that RSI could be used as a tool for designing individually tailored plyometric training, while RSI_mod_ could be used to determine the need of incorporating ballistic-type exercises into training program. Bilateral over unilateral RSI_mod_ is to be preferred for now, as the latter showed poorer reliability, but similar association to approach jump performance compared to the bilateral variant. It has to be noted that RSI outcomes might not add significant additional information (in terms of explaining performance) to DJ and CMJ height alone.
